# Up‐regulation of glycolysis promotes the stemness and EMT phenotypes in gemcitabine‐resistant pancreatic cancer cells

**DOI:** 10.1111/jcmm.13126

**Published:** 2017-02-28

**Authors:** Hengqiang Zhao, Qingke Duan, Zhengle Zhang, Hehe Li, Heshui Wu, Qiang Shen, Chunyou Wang, Tao Yin

**Affiliations:** ^1^ Department of Pancreatic surgery Union Hospital Tongji Medical College Huazhong University of Science and Technology Wuhan China; ^2^ Department of Clinical Cancer Prevention The University of Texas MD Anderson Cancer Center Houston TX USA

**Keywords:** cancer stem cells, epithelial–mesenchymal transition, chemoresistance, reactive oxygen species, gemcitabine, DCLK1

## Abstract

Cancer stem cells (CSCs) and epithelial–mesenchymal transition (EMT)‐type cells are considered as underlying causes of chemoresistance, tumour recurrence and metastasis in pancreatic cancer. We aimed to describe the mechanisms – particularly glycolysis – involved in the regulation of the CSC and EMT phenotypes. We used a gemcitabine‐resistant (GR) Patu8988 cell line, which exhibited clear CSC and EMT phenotypes and showed reliance on glycolysis. Inhibition of glycolysis using 2‐deoxy‐D‐glucose (2‐DG) significantly enhanced the cytotoxicity of gemcitabine and inhibited the CSC and EMT phenotypes in GR cells both *in vitro* and *in vivo*. Intriguingly, the use of the reactive oxygen species (ROS) scavenger *N*‐acetylcysteine (NAC) restored the CSC and EMT phenotypes. H_2_O_2_ produced changes similar to those of 2‐DG, indicating that ROS were involved in the acquired cancer stemness and EMT phenotypes of GR cells. Moreover, doublecortin‐like kinase 1 (DCLK1), a pancreatic CSC marker, was highly expressed and regulated the stemness and EMT phenotypes in GR cell. Both 2‐DG and H_2_O_2_ treatment suppressed DCLK1 expression, which was also rescued by NAC. Together, these findings revealed that glycolysis promotes the expression of DCLK1 and maintains the CSC and EMT phenotypes *via* maintenance of low ROS levels in chemoresistant GR cells. The glycolysis‐ROS‐DCLK1 pathway may be potential targets for reversing the malignant behaviour of pancreatic cancer.

## Introduction

Pancreatic cancer is a highly aggressive malignant disease with a 5‐year survival rate of less than 8% 1. As the most important part of chemotherapy, gemcitabine alone or in combination with other therapeutics has been adopted as the standard chemotherapy for advanced pancreatic cancer, despite a very limited progression‐free survival interval 2, 3. One of the important causes of the disappointing outcome of patients with pancreatic cancer is the acquired drug resistance to gemcitabine 2, 4.

The existence of CSCs is one of the fundamental drivers of chemoresistance in pancreatic cancer 4, 5, 6, 7. CSCs are a subpopulation of tumour cells that are endowed with the capacity for self‐renewal. They are also considered to be an underlying cause of tumorigenesis, tumour recurrence and metastasis 7, 8, 9. Previous studies have demonstrated that GR pancreatic cancer cells have features of CSCs 4, 6, 10, 11. In addition, these chemoresistant cells demonstrate an EMT phenotype, a change from an epithelial “cobblestone” appearance to the spindle‐shaped morphology characteristic of mesenchymal cells, leading to enhanced motility and invasion 6, 10, 12. The CSCs and EMT process appear to be linked and contribute to chemoresistance and promote cancer progression 7. Therefore, elucidating the underlying mechanisms of chemoresistance linked with CSC and EMT phenotypes is of great importance for developing techniques to overcome chemoresistance in pancreatic cancer.

It has been well‐established that cancer cells, unlike normal cells, undergo a metabolic shift known as the Warburg effect, in which glucose metabolism and lactate generation are enhanced even in the presence of oxygen 13. Although aerobic glycolysis is a well‐recognized hallmark of cancer cells, the metabolic signatures particular to chemoresistant cells and their parental cells remain elusive. Recently, it has been reported that cancer cell stemness can be epigenetically regulated by metabolic reprogramming 14. In addition, a recent study demonstrated that radioresistant nasopharyngeal carcinoma CSCs exhibited a greater reliance on glycolysis than did their parental cells and that their stem cell‐like properties could be curbed *via* inhibition of glycolysis 15. Therefore, it is important to elucidate the role of metabolism change in chemoresistance associated with CSC and EMT characteristics in pancreatic cancer cells.

Unlike normal cells, cancer cells maintain high ROS levels and suffer from oxidative stress 16. However, CSCs have lower levels of ROS than do cancer cells in general. In fact, the maintenance of low ROS levels has been found to be essential for maintaining stemness and EMT properties in CSCs 17, 18, 19, 20. Studies have shown that glycolysis accounts for the maintenance of low ROS levels in CSCs 19, 21. ROS have also been reported to link glucose metabolism to CSC and the EMT phenotypes in breast cancer 19. In the light of these observations, we attempt to characterize chemoresistant pancreatic cancer cells from a ROS‐mediated metabolism perspective.

Emerging evidence suggests that DCLK1, a well‐established putative pancreatic CSC marker, regulates the EMT phenotype 22 and facilitates tumour invasion and metastasis 23. However, to the best of our knowledge, studies on the relationship between glycolysis and DCLK1 were not reported. We also explored the roles of glycolysis and ROS involved in the regulation of DCLK1.

In this study, we demonstrated that GR Patu8988 cells were more glycolytic than parental gemcitabine‐sensitive (GS) cells. In addition, glycolysis maintained gemcitabine‐induced CSC and EMT phenotypes *via* maintaining ROS at low levels. Additionally, ROS negatively regulated the expression of DCLK1 which in turn regulated the stemness and EMT properties of GR cells. We conclude that inhibition of glycolysis, up‐regulation of ROS and knockdown of DCLK1 may eradicate CSCs, reverse the EMT phenotype and therefore enhance the chemosensitivity. These findings may open the door for new and innovative therapies for patients with pancreatic cancer.

## Materials and methods

### Cell lines and culture conditions

The human pancreatic cancer line Patu8988 was originated from KeyGEN (China) [Correction added on 14th June 2017, after first online publication: the origin of the cell PATU78988 was incorrect and updated on this version]. GR Patu8988 cells were derived as described previously 10, 12. In short, Patu8988 cells were cultured with increasing concentrations of gemcitabine (Selleck.cn, Shanghai, China) from 20 nM to a final 1000 nM for up to 12 weeks and were finally cultured in 1 μM gemcitabine during multiple passaging. The duration of cultivation in 1 μM gemcitabine was 9 months when the cells completely adapted to the treatment. The resultant cells were termed as GR cells. Both cells were cultured in Dulbecco's modified Eagle's medium (DMEM) (HyClone, Beijing, China) supplemented with 10% foetal bovine serum (Gibco Invitrogen, Grand Island, NY, USA).

### Cell viability assay

This was conducted as described previously 24. Cells (6000/well) were seeded in 96‐well plates overnight. The cells were then treated with different agents for the indicated time. As for the proliferation of the transfected GR cells, cells (2.5 × 10^3^) were seeded and transfected in 96‐well plate. Cell growth was observed for 5 days. MTT (Sigma‐Aldrich, St. Louis, MO, USA) were added and incubated for another 4 hrs. The absorbance was read at 490 nm using a microplate photometer after adding DMSO (Sigma‐Aldrich). Details are shown in supplementary materials and methods of Data S1.

### Western blot analysis

Cells were washed twice with cold PBS and lysed with a radioimmunoprecipitation assay lysis buffer (Beyotime Biotechnology, Shanghai, China) at 4°C for 30 min. The total protein was extracted, and the concentration of each sample was determined using a BCA protein assay kit (Beyotime) according to the manufacturer's instructions. Equal amounts of protein were subjected to sodium dodecyl sulphate–polyacrylamide gel electrophoresis and transferred to polyvinylidene fluoride membranes (Millipore, Billerica, MA, USA) which were then blocked with 5% non‐fat milk powder dissolved in Tris‐buffered saline with Tween‐20 (TBST) for 1 hr and incubated with primary antibodies over night at 4°C. The membranes were washed with TBST three times (10 min. each), incubated with secondary horseradish peroxidase‐coupled antibodies (Aspen, Wuhan, China) and visualized using ECL substrate (ThermoFisher, Waltham, MA, USA). The antibodies were provided in the supplementary materials and methods Data S1.

### Quantitative real‐time PCR assay

Cellular RNA was extracted using TRIzol (Invitrogen, Carlsbad, CA, USA). cDNA was obtained by reverse transcription with 0.5 μg of RNA with PrimeScript RT Master Mix (Takara Bio, Kusatsu, Shiga, Japan). Quantitative real‐time PCR (qRT‐PCR) was performed using a quantitative SYBR Green PCR Kit (Takara Bio). Each sample was set up in triplicate wells. The mRNA levels of the targeted genes were expressed with the 2^−ΔΔCT^ method and normalized to GAPDH. Primer sequences are listed in Table S1.

### Migration and invasion assays

Cells (5 × 10^4^ cells) in 200 μL DMEM plus 0.1% foetal bovine serum (FBS) were plated into the upper compartment of a Transwell chamber (Corning, Costar, NY, USA) coated with ECM gel (Sigma‐Aldrich) or left uncoated. The lower chamber was filled with 700 μL DMEM plus 20% FBS. After the cells were cultured for 24 hrs, the cells in the upper chamber were removed with a cotton swab. The invaded cells were fixed with 4% paraformaldehyde and stained with 0.1% crystal violet for visualization. Cells were counted in five respective fields at 200× magnification using a microscope (Olympus, Tokyo, Japan).

### Flow cytometric analysis

Pancreatic CSC surface markers CD133 and CD24 and ROS levels were detected using flow cytometry as described previously 25. Cells were stained with fluorescein isothiocyanate‐conjugated CD24 antibody (BD Pharmingen, San Diego, CA, USA) or phycoerythrin‐conjugated CD133 antibody (Miltenyi Biotec, Bergisch Gladbach, Germany) or isotype control IgG (Biolegend, San Diego, CA, USA) and were analysed using a flow cytometer (BD, Biosciences, Franklin Lakes, NJ, USA). Levels of intracellular ROS were determined using an ROS assay kit (Beyotime) according to the manufacturer's instructions. Details are shown in supplementary materials and methods of Data S1.

### Sphere‐formation assay

Cells (5 × 10^3^) with different treatments were cultured in serum‐free Dulbecco's modified Eagle's medium (DMEM)/Ham's nutrient mixture (F12) (1:1) medium (Invitrogen) supplemented with 20 ng/ml of epithelial growth factor (PeproTech, Rocky Hill, NJ, USA) and 10 ng/ml of basic fibroblast growth factor (PeproTech) for 14 days. Twice a week, half of the culture medium was replaced and the medium was supplemented. Spheres larger than 50 μm were counted using a microscope (Olympus) 6.

### Small interfering RNA‐mediated knockdown of DCLK1

Small interfering RNA (siRNA) (RiboBio Co. Guangzhou, China) sequence targeting the coding region of DCLK1 (siDCLK1#1: CAGAGGTGCGAGAGAATAA and siDCLK1#2: CTGGAAAGATAAAGAAGCA) and negative control siRNA (siNC) not matching any of the human genes were obtained. Knockdown of DCLK1 was performed by transfecting cells with Lipofectamine 2000 (Invitrogen) according to the manufacturer's instructions. The efficiency of the transfection was confirmed by Western blot and qRT‐PCR analyses.

### Glucose uptake and lactate production assays

Glucose uptake and lactate production were performed according to previously published methods 26. The complete medium was replaced with a glucose‐free medium and incubated for 2 hrs. Cells were then incubated with the fluorescence‐labelled glucose analogue (2‐(*N*‐(7‐nitrobenz‐2‐oxa‐1,3‐diazol‐4‐yl)amino)‐2‐deoxyglucose) 2‐NBDG (Cayman Chemical, Ann Arbor, MI, USA) with a final concentration of 10 μM for 30 min. at 37°C. The uptake of 2‐NBDG was analysed with flow cytometry and fluorescence microscope. For assessment of lactate production, cells (2.5 × 10^5^) were cultured in complete medium. After 36 hrs, the supernant was collected and centrifugated. Lactate production was assessed using a lactic acid assay kit (Nanjing Jiancheng Bio. Nanjing, China) according to the manufacturer's protocol and was corrected for the total protein in each sample.

### Animal experiment

All animal experiments were handed in compliance with the Institutional Animal Care and Use Committee of Tongji Medical College of Huazhong University of Science and Technology. For subcutaneous tumour formation, GS or GR cells with different treatments were subcutaneously injected into the right flank of nude mice. In addition, an orthotopic transplantation pancreatic cancer model was performed to assess liver metastasis as described previously 27. The detailed treatment protocols are shown in supplementary materials and methods of Data S1.

### Statistical analysis

All data were analysed using GraphPad Prism 5.0 statistical software (San Diego, CA, USA). Mann–Whitney *U*‐test was used to calculate the significant difference between two groups. For data set containing more than two groups, one‐way anova with Bonferroni post‐tests was carried out. Two‐way anova was used to determine differences over different drug treatments. Results were expressed as means ± S.E.M. *P* values <0.05 were considered statistically significant.

## Results

### GR cells display enhanced aerobic glycolysis and glycolytic dependency

**Figure 1:**
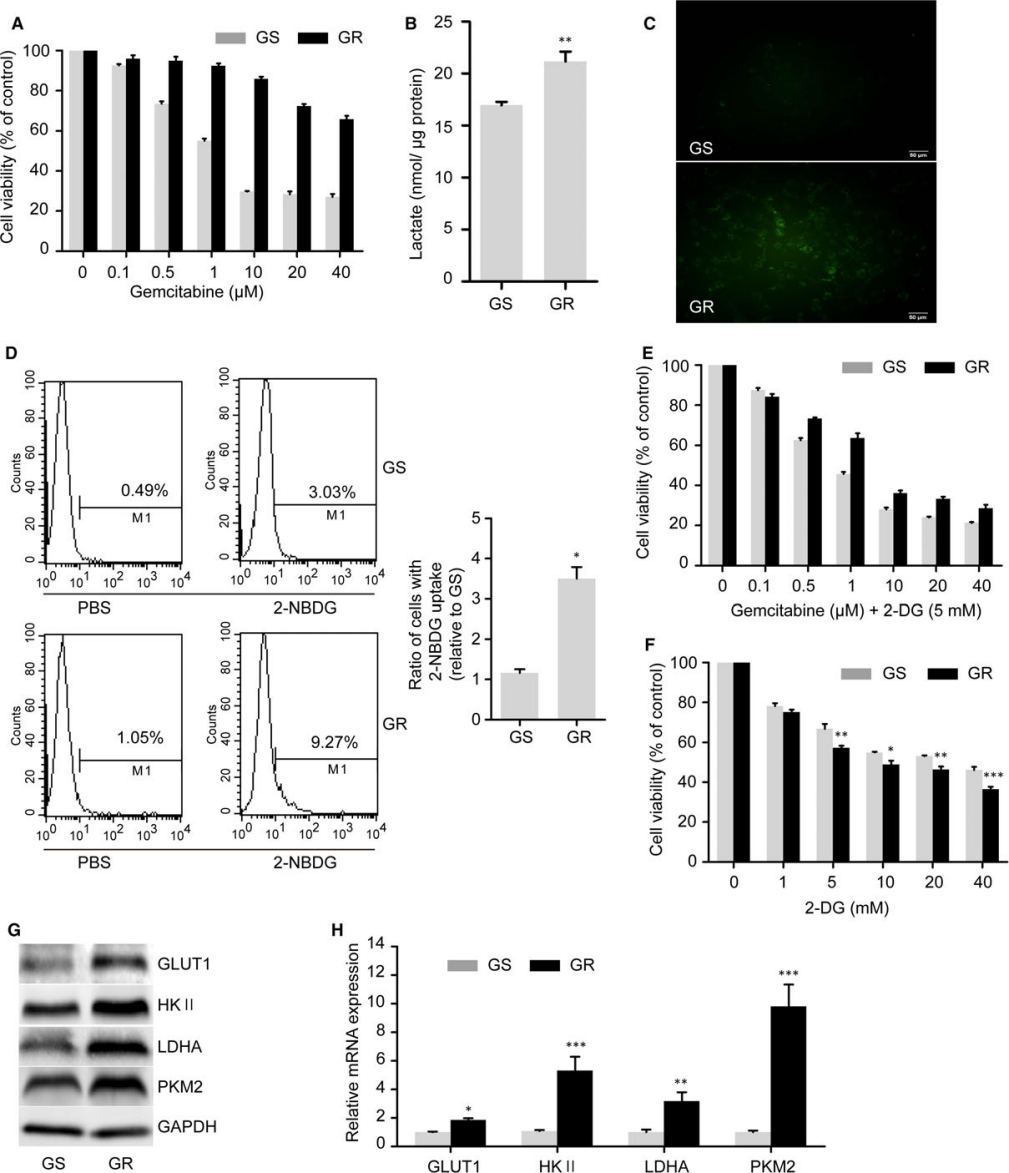
GR cells are more glycolytic than parental GS cells. (**A**) GR cells were established as described previously. Cell viability was measured using an MTT assay. (**B**) GR cells had significantly higher levels of lactate production than did GS cells. (**C**–**D**) 2‐NBDG uptake in GS and GR cells. GS and GR cells were treated with 2‐NBDG or PBS for the indicated time. The fluorescence intensity which indicated the uptake of 2‐NBDG was measured by fluorescence microscope (**C**)**,** and the ratio of the cells with 2‐NBDG uptake was measured using flow cytometry and then quantified (**D**). Scale bar, 50 μm. (**E**–**F**) Cell viability of GS and GR cells towards gemcitabine + 2‐DG or 2‐DG alone. (**E**) Both types of cells were treated with increasing concentrations of gemcitabine combined with 5 mM 2‐DG for 48 hrs. (**F**) Cell viability of GS and GR cells towards increasing concentrations of 2‐DG for 48 hrs. (**G**) Glycolysis‐associated enzymes and proteins were measured by Western blot analysis. GAPDH was used as a loading control. (**H**) mRNA expression levels of GLUT1, HK‐II, LDHA and PKM2 were significantly higher in GR cells than in GS cells using qRT‐PCR analysis. The data shown are representative of three independent experiments. Bars represent means ± S.E.M. *P < 0.05; **P < 0.01; ***P < 0.001 compared with GS.

Cell viability assays showed that the half‐maximal inhibitory concentrations (IC50) of gemcitabine for GR and GS cells were 134.3 μM and 2.8 μM, respectively (Fig. 1A). GR cells, therefore, showed 48‐fold greater resistance to gemcitabine than did GS cells.

We next compared metabolic changes in GR cells and GS cells. GR cells generated significantly larger amounts of lactate than did parental cells (Fig. 1B). Using (2‐(*N*‐(7‐nitrobenz‐2‐oxa‐1,3‐diazol‐4‐yl)amino)‐2‐deoxyglucose) (2‐NBDG), a fluorescent deoxyglucose analogue, to monitor glucose uptake, we found that 2‐NBDG uptake in GR cells was significantly higher than that in GS cells (Fig. 1C and D).

To further examine the dependence of pancreatic cancer cells on glycolysis for survival, we evaluated cell viability upon addition of 2‐DG, an inhibitor of glycolysis. The IC_50_ of gemcitabine decreased from 134.3 μM to 3.9 μM in GR cells and from 2.8 μM to 1.4 μM in GS cells after the introduction of 2‐DG (Fig. 1A and E). Furthermore, cell viability decreased significantly more in GR cells than in GS cells after treatment with 2‐DG alone (Fig. 1F), further indicating that GR cells were more dependent on glycolysis for survival.

In accordance with our finding that GR cells have high glycolytic flux, assays of both protein and mRNA expressions showed that the levels of the glycolytic proteins and enzymes glucose transporter 1 (GLUT1), hexokinase‐II (HK‐II), lactate dehydrogenase A (LDHA) and pyruvate kinase M2 (PKM2) were significantly higher in GR cells (Fig. 1G and H).

### GR cells demonstrate the CSC and EMT phenotypes compared with GS cells

**Figure 2:**
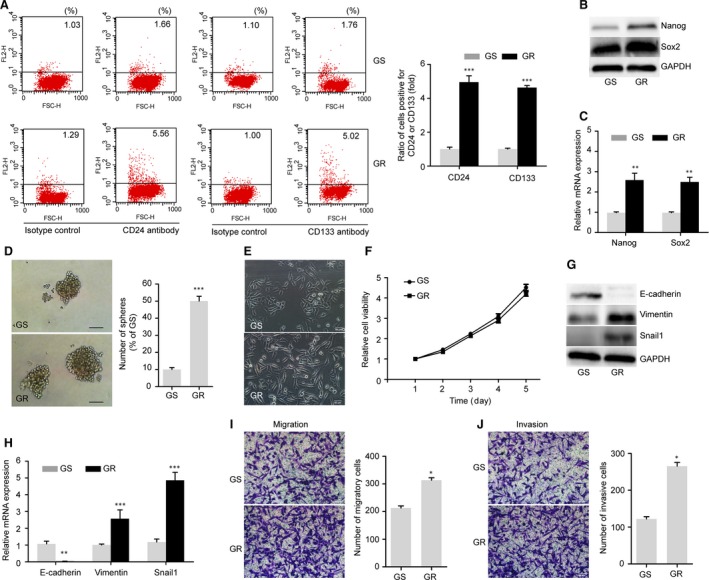
GR cells demonstrate the CSC and EMT phenotypes compared with GS cells. (**A**–**D**) GR cells exhibited enhanced stemness properties compared with their GS cells. (**A**) Representative flow cytometric analysis using conjugated antibodies against the pancreatic cancer stem cell markers CD133 and CD24 (left) and quantification of the assay (right). (**B**–**C**) GR cells had higher expression levels of the pluripotency markers Nanog and Sox2 in both Western blot and qRT‐PCR analyses. (**D**) Sphere‐formation assay of GS and GR cells. Representative images of spheres (left) and quantification of the assay (right). Scale bar, 50 μm. (**E**–**J**) GR cells exhibited more EMT characteristics than did GS cells. (**E**) Morphology of GS and GR cells (×200). (**F**) The proliferation of GS and GR cells was measured using MTT assays. Cells (2 × 10^3^) were seeded in 96‐well plate, and the growth was observed for 5 days. (**G**–**H**) Western blot and qRT‐PCR analyses showed the down‐regulation of E‐cadherin and the up‐regulation of Vimentin and Snail1 in GR cells. (**I**–**J**) GR cells exhibited increased migratory and invasive capacities. GS and GR cells (5 × 10^4^ cells) were allowed to migrate or invade Matrigel‐coated or uncoated Transwell chambers for 24 hrs. Representative images (×200) of the cells that migrated or invaded the membrane of the chamber and were counted as described in the ‘Materials and Methods’. Data are expressed as means ± S.E.M. of three independent experiments. *P < 0.05; **P < 0.01; ***P < 0.001. GAPDH was used as a loading control.

We found that GR cells displayed a CSC‐like phenotype (Fig. 2A–D). Flow cytometric analysis using antibodies against pancreatic CSC surface markers showed that GR cells had an obviously higher expression of the stemness markers CD24 and CD133 on their cell surfaces than did GS cells (Fig. 2A). Intriguingly, both Western blot and qRT‐PCR analyses indicated that the pluripotency markers Nanog and Sox2 were expressed at higher levels in GR cells than in GS cells (Fig. 2B and C). In addition, the sphere‐formation assay showed that GR cells formed more and larger spheres than did GS cells (Fig. 2D). Taken together, these findings suggest that chemoresistant GR cells possess enhanced stem‐like cell characteristics than their GS parental cells.

GR cells, which exhibit a loss of cell–cell adhesion, increased formation of pseudopodia, and spindle‐shaped morphology, were morphologically distinct from GS cells (Fig. 2E). The MTT assay showed that there was no obvious change in the proliferation between GS and GR cells (Fig. 2F). We next investigated markers of EMT. Assays of both protein and mRNA levels showed that GR cells had lower expression of the epithelial marker E‐cadherin and higher expression of the mesenchymal marker Vimentin and the transcriptional factor zinc finger protein SNAI1 (Snail 1) than did GS cells (Fig. 2G and H). One of the important characteristics of mesenchymal‐like cells is their enhanced capacity for migration and invasion 10. Consistent with our morphological observations, we found that GR cells demonstrated significantly greater capacity for migration and invasion than did GS cells (Fig. 2I and J). These observations suggest that GR cells possess the EMT property, becoming more invasive *in vitro*. To further observe whether the biological characteristics of GR cells were reversible, we cultured GR cells in gemcitabine‐free medium up to 7 days. Interestingly, these GR cells still showed enhanced chemoresistance and invasive and self‐renewal capacities compared with their parental GS cells (data not shown), indicating that GR cells underwent stable biological transformation during the long‐term induction.

### Glycolysis regulates the gemcitabine‐induced CSC and EMT phenotype *via* maintaining low ROS levels

**Figure 3:**
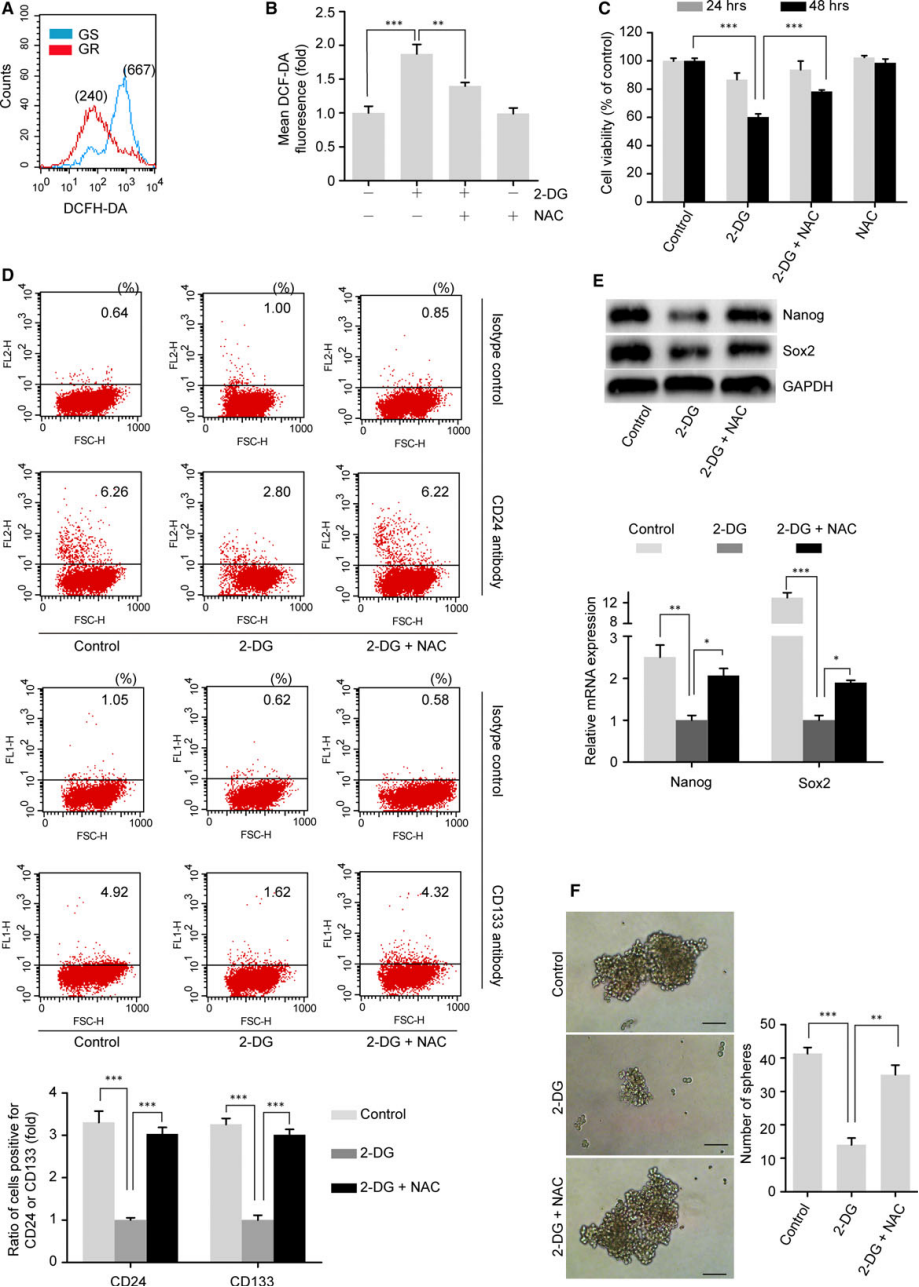
Glycolysis regulates the CSC property *via* maintaining low levels of ROS in GR cells. (**A**) The ROS levels of GS and GR cells were measured using flow cytometry with a DCFH‐DA probe. The numbers in parentheses indicate the mean fluorescent intensity. (**B**) ROS levels in GR cells treated with or without 2‐DG (5 mM) in the presence or absence of NAC (5 mM) for 12 hrs. (**C**) Cell viability of GR cells treated with or without 2‐DG (5 mM) in the presence or absence of NAC (5 mM) for 24 hrs or 48 hrs. (**D**–**F**) The introduction of NAC rescued the inhibition of stemness caused by 2‐DG in GR cells. GR cells were treated with or without 2‐DG (5 mM) in the presence or absence of NAC (5 mM) for 24 hrs. (**D**) Flow cytometry was used to assess the expression of CD133 and CD24 in GR cells treated as indicated. Representative flow cytometric analysis (upper) and quantification of the assay (lower). (**E**) Western blot (upper) and qRT‐PCR (lower) analyses were performed to analyse the pluripotency markers Nanog and Sox2 in GR cells treated as indicated. (**F**) Sphere‐formation assay of GR cells with different treatments. The treated cells (5 × 10^3^ cells) were allowed to grow in serum‐free medium for 14 days. Scale bar, 50 μm. The data shown are from three parallel experiments. Bars represent means ± S.E.M. *P < 0.05; **P < 0.01; ***P < 0.001. GAPDH was used as a loading control.

To further elucidate the role of ROS in the acquired stemness and EMT phenotypes of GR cells, we used flow cytometry to assess ROS levels. We found that ROS levels were obviously lower in GR cells than in GS cells (Fig. 3A), indicating that the gemcitabine‐induced stemness and EMT phenotypes may be associated with intrinsically low ROS levels. Because it has been reported that 2‐DG treatment can inhibit glycolysis and induce cytotoxicity *via* oxidative stress and that 2‐DG's cytotoxicity can be suppressed by the antioxidant NAC 28, 29, 30, 31, we first investigated these effects of 2‐DG treatment. As we expected, 2‐DG treatment induced ROS production, which could be inhibited by treatment with NAC (Fig. 3B). In addition, cell viability assay showed that the viability was not significantly affected within 24 hrs in GR cells treated with or without 2‐DG in the presence or absence of NAC. However, NAC inhibited the cytotoxicity induced by treatment with 2‐DG at 48 hrs (Fig. 3C). In the light of these observations, we further investigated the effect of oxidative stress induced by glycolytic inhibition on the stemness and EMT phenotypes in GR cells.

GR cells were treated with or without the glycolytic inhibitor 2‐DG in the presence or absence of NAC for the indicated time. We found that 2‐DG effectively decreased the expression of the pancreatic cancer stemness markers CD24 and CD133 (Fig. 3D) and reduced the levels of pluripotency markers Nanog and Sox2 in both protein and mRNA levels (Fig. 3E). 2‐DG treatment also suppressed the sphere‐forming ability of GR cells (Fig. 3F). However, compared with cells treated with 2‐DG alone, cells treated with a combination of 2‐DG and NAC exhibited increased levels of CD24 and CD133 (Fig. 3D), higher Nanog and Sox2 protein levels (Fig. 3E, upper), increased mRNA expression of Nanog and Sox2 (Fig. 3E, lower) and enhanced sphere‐formation ability (Fig. 3F).

**Figure 4:**
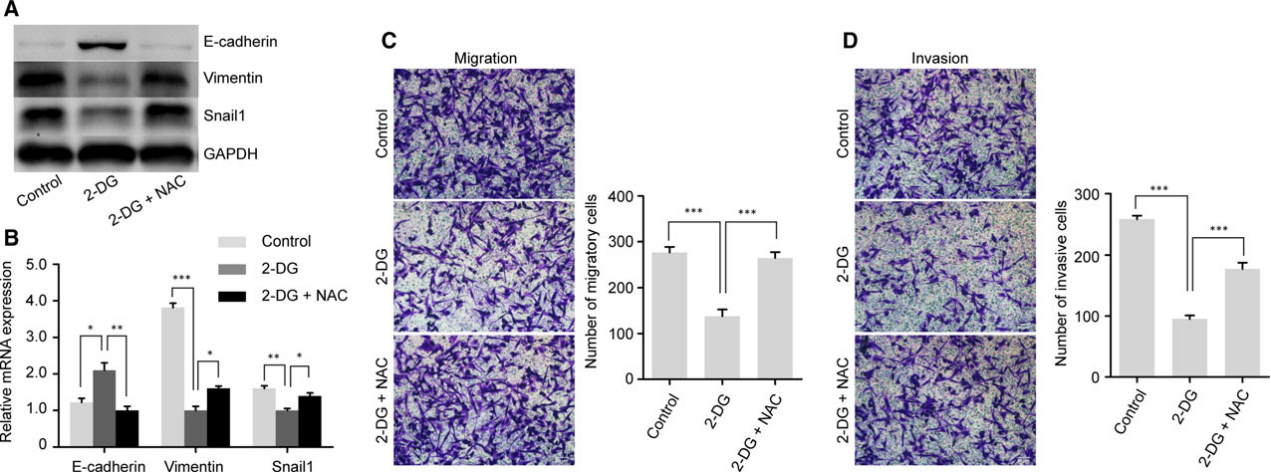
Role of glycolysis in the maintenance of EMT phenotype in GR cells. (**A**–**D**) The introduction of NAC restored the EMT phenotype inhibited by 2‐DG in GR cells. (**A**–**B**) The protein and mRNA expression levels of E‐cadherin, Vimentin and Snail1 were measured using Western blot and qRT‐PCR analyses in GR cells treated as indicated. (**C**–**D**) Transwell assays depicted the migratory and invasive abilities in GR cells treated as in Fig. 3D for 24 hrs. Magnification, ×200. The data shown are from three parallel experiments. Bars represent means ± S.E.M. *P < 0.05; **P < 0.01; ***P < 0.001. GAPDH was used as a loading control.

In addition, both Western blot and qRT‐PCR analyses indicated that E‐cadherin expression was up‐regulated and that Vimentin and Snail1 were down‐regulated after 2‐DG treatment (Fig. 4A and B). Additionally, GR cells treated with 2‐DG showed a significantly inhibited capacity for migration and invasion (Fig. 4C and D). However, NAC combined with 2‐DG treatment also restored the EMT phenotype as indicated by the significant down‐regulation of E‐cadherin expression and up‐regulation of Vimentin and Snail1 expression compared with the levels in GR cells treated with 2‐DG alone (Fig. 4A and B). Finally, the increased capacity for migration and invasion that had been inhibited in GR cells by 2‐DG treatment was also restored after the introduction of NAC (Fig. 4C and D).

**Figure 5:**
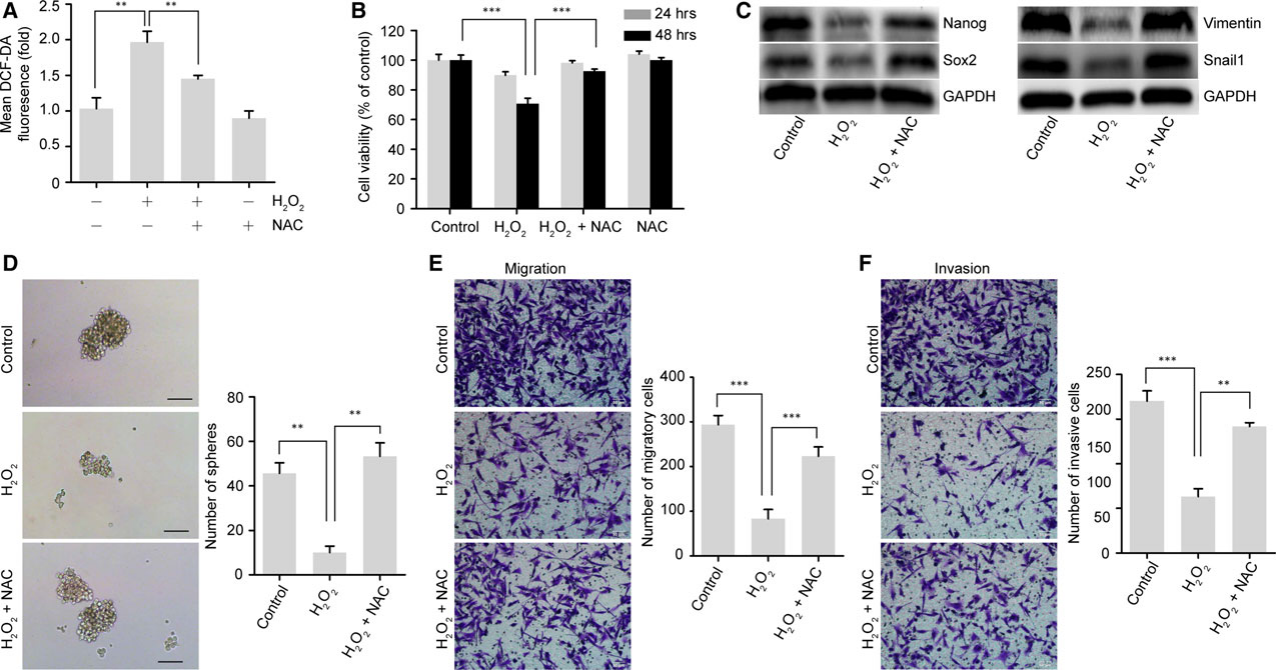
ROS are involved in the regulation of gemcitabine‐induced CSC and EMT phenotypes. (**A**) ROS levels in GR cells left untreated or treated with H_2_O_2_ (200 μM), NAC (5 mM) or H_2_O_2_ + NAC for 6 hrs were assessed using flow cytometry. (**B**) Cell viability of GR cells treated with or without H_2_O_2_ (200 μM) in the presence or absence of NAC (5 mM) for 24 hrs or 48 hrs. (**C**) NAC suppressed H_2_O_2_‐induced down‐regulation of the stem cell markers Nanog and Sox2 and mesenchymal markers Vimentin and Snail1 in GR cells. GR cells were left untreated, or treated with H_2_O_2_ (200 μM), or H_2_O_2_ + NAC (5 mM) for 24 hrs. GAPDH was used as a loading control. (**D**) Comparison of sphere‐formation ability in GR cells treated as in (**C**). The treated cells were cultured in serum‐free medium for 14 days. Scale bar, 50 μm. (**E**–**F**) Migratory and invasive capacities of GR cells treated as in (**C**). Data are expressed as means ± S.E.M. of three independent experiments. **P < 0.01; ***P < 0.001.

To further investigate the role of ROS in the maintenance of the CSC and EMT phenotypes, we introduced exogenous H_2_O_2_ to induce an increase in ROS levels comparable to that induced by 2‐DG in GR cells (Fig. 5A). We also assessed the oxidative stress induced by H_2_O_2_ treatment on cell viability. Similar to 2‐DG treatment, the cell viability was not significantly affected within 24 hrs in GR cells. NAC also inhibited the cytotoxicity induced by treatment with H_2_O_2_ at 48 hrs (Fig. 5B). Like 2‐DG treatment, H_2_O_2_ treatment decreased the protein expression of the pluripotency markers Nanog and Sox2 and the EMT markers Vimentin and Snail1. However, the addition of NAC blocked the down‐regulation of these proteins (Fig. 5C). We also found that H_2_O_2_ significantly inhibited sphere formation and reduced the migratory and invasive capacity of GR cells. NAC prevented the H_2_O_2_‐induced loss of the CSC and EMT phenotypes (Fig. 5D–F). All these results suggest that glycolysis maintains the gemcitabine‐induced CSC and EMT phenotypes *via* down‐regulating ROS production in GR cells.

### The glycolysis‐ROS‐DCLK1 pathway accounts for the maintenance of gemcitabine‐induced CSC and EMT phenotypes

**Figure 6:**
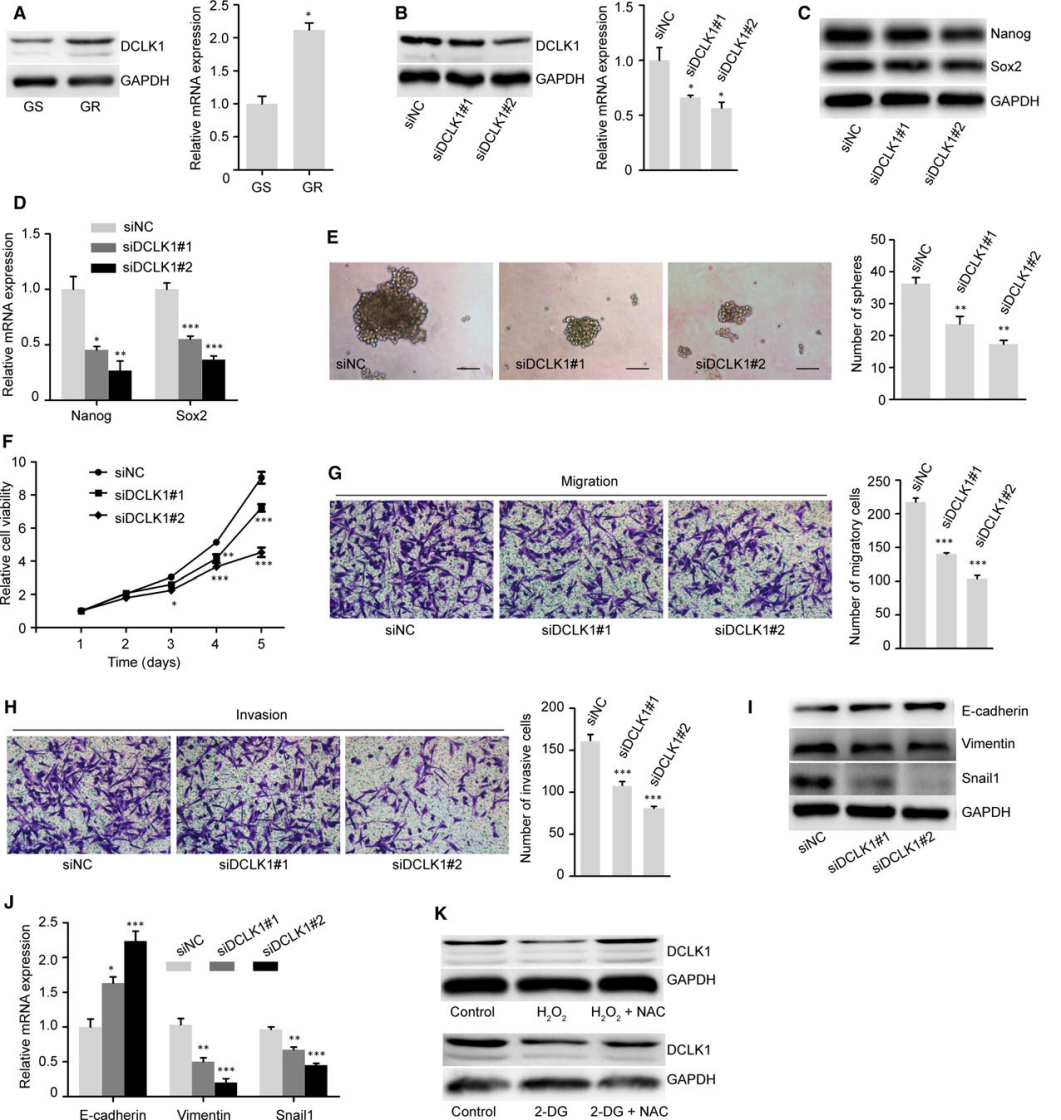
The glycolysis‐ROS‐DCLK1 pathway is associated with gemcitabine‐induced CSC and EMT phenotypes. (**A**) Expression of DCLK1 was measured by Western blot (left) and qRT‐PCR (right) analyses in GS and GR cells. (**B**) Transfection with two small interfering DCLK1 RNA (siDCLK1#1 and siDCLK1#2) decreased the protein (left) and mRNA (right) expression of DCLK1 in GR cells compared with negative control siRNA (siNC) 48 hrs post‐transfection. (**C**–**D**) Knockdown of DCLK1 down‐regulated protein and mRNA expression levels of the pluripotency markers Nanog and Sox2 in GR cells 48 hrs post‐transfection. (**E**) siDCLK1 significantly suppressed sphere formation in GR cells compared with siNC 48 hrs post‐transfection. Scale bar, 50 μm. (**F**) Silencing DCLK1 expression decreased the proliferation of GR cells using MTT assay. (**G**–**H**) siDCLK1 significantly inhibited migration and invasion in GR cells compared with siNC 48 hrs post‐transfection. Magnification, ×200. (**I**–**J**) siDCLK1 up‐regulated the expression of E‐cadherin and down‐regulated the expression of Vimentin and Snail1 compared with siNC in protein and mRNA levels. (**K**) DCLK1 expression was measured by Western blot analysis in GS and GR cells treated as previous description. GAPDH was used as a loading control. The data shown are representative of three independent experiments. Bars represent means ± S.E.M. *P < 0.05; **P < 0.01, ***P < 0.001 compared with siNC.

We found that GR cells had a higher expression level of DCLK1 than did GS cells in protein and mRNA levels (Fig. 6A). To confirm the involvement of DCLK1 in the maintenance of stemness and the EMT phenotypes in GR cells, we transfected siRNA‐targeting DCLK1 (siDCLK1#1 and siDCLK1#2) into GR cells. The successful transfection of siDCLK1 was verified by a reduction in the expression of both the DCLK1 protein (Fig. 6B, left) and DCLK1 mRNA (Fig. 6B, right) expressions compared with negative siRNA controls (siNC). Both protein and mRNA levels of the pluripotency markers Nanog and Sox2 were decreased after siDCLK1 transfection (Fig. 6C and D). In addition, the sphere‐forming ability of GR cells was significantly suppressed by siDCLK1 transfection (Fig. 6E). The MTT assay showed that silencing DCLK1 inhibited the proliferation of GR cells (Fig. 6F). DCLK1 knockdown also inhibited EMT phenotype. The migratory capability and invasive capability of GR cells were reduced after siDCLK1 transfection (Fig. 6G and H). In addition, the protein expression and mRNA levels of the epithelial marker E‐cadherin were up‐regulated, while the mesenchymal marker Vimentin and the transcription factor Snail1 were down‐regulated after siDCLK1 transfection (Fig. 6I and J).

We next examined the roles of glycolysis and ROS in DCLK1 expression. H_2_O_2_ administration decreased the expression of DCLK1 in GR cells; the introduction of NAC restored DCLK1 expression (Fig. 6K), indicating the involvement ROS in DCLK1 expression. Interestingly, the glycolysis inhibitor 2‐DG decreased the expression of DCLK1 in GR cell, and this expression was also recovered by the introduction of NAC (Fig. 6K). Together, these data suggest that glycolysis regulates DCLK1 expression *via* down‐regulating ROS production in GR cells exhibiting the CSC and EMT phenotypes.

### Glycolysis promotes tumorigenesis and metastasis *in vivo*

**Figure 7:**
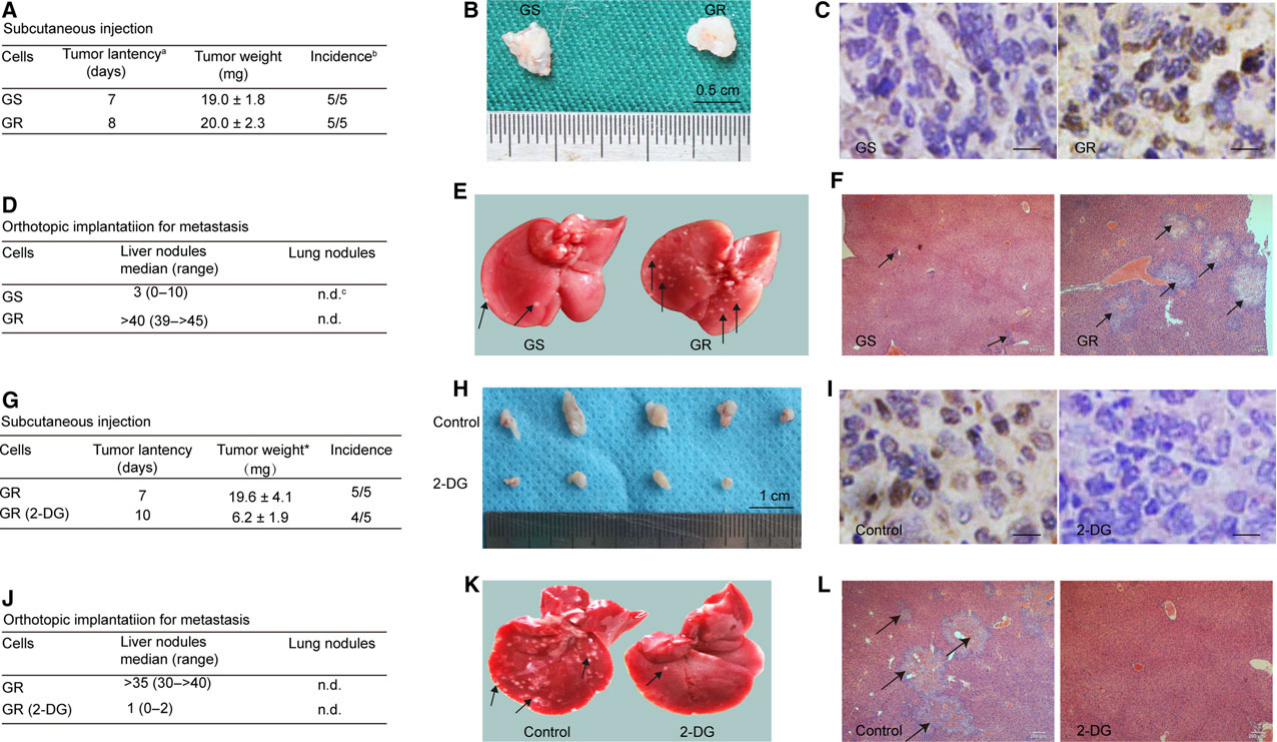
Glycolysis is involved in the tumorigenesis and metastasis *in vivo*. (**A**–**C**) The comparison of tumorigenicity between GS and GR cells. (**A**) GS and GR cells (5 × 10^6^ each) were subcutaneously injected into the right flank of mice (5 mice/group). Tumour weight was expressed as means ± S.E.M. ^a^Time between tumour cell injection and detection of a palpable tumour in mice. ^b^Number of mice with tumour / number of inoculated mice. (**B**) Representative photographs of tumours at 21 days. (**C**) Immunohistochemistry staining of DCLK1 from tumours sections. Scale bar, 30 μm. (**D**–**F**) The comparison of metastatic ability between GS and GR cells. (**D**) GS and GR cells (5 × 10^6^ each) were orthotopically transplanted into the head of mouse pancreas. Mice were killed after 4 weeks. ^c^Not detected at 4 weeks from injection of tumour cells. (**E**) Macroscopic findings of the resected livers in mice. (**F**) H&E staining (×40) of the resected livers. The blank arrows represent the metastatic nodules. (**G**–**I**) 2‐DG treatment inhibited the tumorigenesis of GR cells. (**G**) GR cells left untreated or treated with 2‐DG for 24 hrs (5 × 10^6^ each) were subcutaneously injected into the right flank of nude mice (5 mice/group). *P < 0.05. (**H**) Representative photographs of tumours at 21 days. (**I**) Immunohistochemistry staining of DCLK1 from tumour sections. Scale bar, 30 μm. (**J**–**L**) 2‐DG treatment inhibited the metastasis of GR cells. (**J**) GR cells left untreated or treated with 2‐DG for 24 hrs (5 × 10^6^ each) were orthotopically transplanted into the head of mouse pancreas. The median values of metastatic liver nodules at 28 days were shown. (**K**–**L**) macroscopic and microscopic (×40) findings of the resected livers in mice. The metastatic nodules were indicated by blank arrows.

We further investigate the role of glycolysis in tumour formation and metastasis *in vivo*. We did not observe significant differences in tumour latency, tumour weight (19.0 ± 1.8 mg *versus* 20.0 ± 2.3 mg, *P* > 0.05) and tumour incidence in mice inoculated with GS or GR cells (Fig. 7A and B). However, tumours originated from GR cells had enhanced staining for DCLK1 (Fig. 7C). In addition, in our orthotopic transplantation pancreatic cancer model, GR cells gave rise to a 13‐fold increase in formation of metastatic liver nodules compared with GS cells (Fig. 7D–F). These findings suggest that GR cells are more invasive and acquired increased metastatic ability *in vivo*.

To further illuminate the role of glycolysis in tumorigenesis and metastasis, we treated GR cells with or without 2‐DG. GR cells formed tumour nodules in 5 of 5 injected mice, whereas 2‐DG‐treated GR cells formed tumour nodules in 4 of 5 injected mice (Fig. 7G and H). In addition, 2‐DG‐treated cells formed reduced tumour weight (6.2 ± 1.8 mg *versus* 19.58 ± 4.1 mg, *P* < 0.05) compared with GR cells (Fig. 7G). Furthermore, tumours formed by 2‐DG‐treated cells showed reduced staining of DCLK1 (Fig. 7I). Moreover, 2‐DG‐treated cells resulted in obviously decreased liver metastases (Fig. 7J–L). These observations suggest that glycolysis is involved in the tumorigenesis and metastasis of GR cells *in vivo*.

## Discussion

Acquired resistance to chemotherapeutic agents is an obstacle in treating pancreatic cancer 2. Accumulating evidence has shown that a small population of pancreatic cancer cells with stem cell‐like features are responsible for chemoresistance, tumorigenesis, progression and metastasis 7, 32. In addition, EMT‐type cells are also believed to play critical roles in drug resistance in pancreatic cancer 33. In this study, we for the first time demonstrated the functional role of glycolysis in the maintenance of chemoresistance, the CSC and EMT phenotypes of GR pancreatic cancer cells *via* down‐regulation of ROS levels.

It has been reported that energy metabolism plasticity determines stemness potential 34. Cancer cells undergo a metabolic shift in which they become more dependent on glycolysis for survival than are normal cells 13. Therefore, it is reasonable to speculate that glycolysis plays a role in the acquired stemness phenotype of pancreatic cancer cells. Shen *et al*. demonstrated that radioresistant nasopharyngeal carcinoma cells exhibited a greater reliance on glycolysis than their parental cells and the stem cell‐like properties could be curbed *via* inhibition of glycolysis 15. We found that GR cells showed a higher glycolysis flux than their parental cells. We also found that the glycolytic inhibitor 2‐DG significantly reinforced the cytotoxicity of gemcitabine towards GR cells. Importantly, 2‐DG treatment led to the inhibition of CSC and the EMT phenotypes in GR cells both *in vitro* and *in vivo*. Therefore, a combination of a glycolytic inhibitor with conventional chemotherapeutic drugs might effectively enhance chemosensitivity in chemoresistant pancreatic cancer cells and help prevent recurrence and metastasis after chemotherapy 21.

ROS, as an intracellular messenger, play various roles in regulating important cell functions. The connection between ROS homeostasis and energy metabolism is complex 16. ROS have been reported to link glucose metabolism to the CSC and EMT phenotypes in breast cancer 19. Moreover, evidence suggests that 2‐DG treatment can cause oxidative stress in cancer cells 30, 31. In the light of these findings, we have elucidated the effects of oxidative stress in the inhibition of stemness and EMT phenotypes in chemoresistant pancreatic cancer cells. As expected, the introduction of the ROS scavenger NAC restored the 2‐DG‐induced loss of CSC and EMT phenotypes in GR cells. Furthermore, the application of NAC also recovered the H_2_O_2_‐induced loss of the CSC and EMT phenotypes in GR cells, further demonstrating that glycolysis is responsible for the maintenance of the gemcitabine‐induced CSC and EMT phenotypes *via* maintaining ROS at relatively low levels.

Previous studies have shown that CSCs possess relatively low intracellular ROS levels 17, 35, 36. In line with these studies, we found that GR cells also displayed lower ROS levels than did their parental GS cells. The elevation of ROS may lead to the differentiation of CSCs and the loss of stem cell markers 37. Therefore, disrupting ROS homeostasis and promoting ROS production may be an effective approach to eradicating CSCs and reversing chemoresistance of pancreatic cancer.

Our study has further elucidated the underlying mechanisms by which ROS affect the stemness in GR cells. We focused on DCLK1 because it is a newly identified CSC marker which is functionally involved in driving cancer carcinogenesis and cancer progression 38. One novel findings of this study is that DCLK1 is highly expressed in GR cells; this finding is consistent with the finding that GR cells have stem cell‐like features. We also found that tumours formed by GR cells had obviously increased staining of DCLK1 compared with their GS parental cell. In addition, we found that DCLK1 was responsible for the maintenance of stemness and the EMT phenotypes in pancreatic cancer cells, which was in line with a previous study 39. Additionally, our results showed that H_2_O_2_ or 2‐DG treatment decreased DCLK1 expression, which was restored by NAC, indicating that ROS negatively regulate the expression of DCLK1 in GR cells. Furthermore, tumours formed by GR cells treated with 2‐DG showed a decreased staining for DCLK1. Collectively, these findings reveal that glycolysis‐related ROS regulate the CSC and EMT phenotypes by targeting DCLK1 in GR cells, suggesting that DCLK1 is a promising target in treating pancreatic cancer.

In our *in vivo* study, we did not observe the differences of tumour growth between GS and GR cells although GR cells showed increased expression of stem cell‐like markers and sphere‐formation ability, indicating that other mechanisms other than glycolysis may be involved in the regulation of tumorigenesis *in vivo*. The present observation needs further investigation for the complex mechanisms involved in chemoresistance in cancer cells.

In conclusion, our data provide the first evidence that glycolytic signalling plays a functional role in the maintenance of CSC and the EMT phenotypes *via* down‐regulation of ROS production and up‐regulation of DCLK1 expression in GR pancreatic cancer cells. Therefore, inhibition of glycolysis signalling, up‐regulation of ROS generation and/or silencing of DCLK1 may be effective approaches to eliminating CSCs, reversing the EMT phenotype and thus eradicating chemoresistant cancer cells to improve the prognosis of patients with pancreatic cancer.

## Conflicts of interest

The authors declare no potential conflicts of interest.

## Authors’ contributions

H. Zhao, Q. Duan, T. Yin and C. Wang involved in conception and design. H. Zhao, Q. Duan, Z. Zhang, H. Li and Q. Shen involved in acquisition of data. H. Zhao, Q. Duan, Z. Zhang, H. Wu, T. Yin and C. Wang involved in analysis and interpretation of data. H. Zhao, Q. Duan, H. Li, H. Wu, Q. Shen, T. Yin and C. Wang involved in writing, review and/or revision of the manuscript. H. Zhao and Q. Duan involved in administrative, technical or material support. T. Yin and C. Wang involved in study supervision.

## Supporting information


**Table S1** PCR primer sequences.

**Data S1** Supplementary Materials and Methods.
