# Supplementary material for: Up‐regulation of glycolysis promotes the stemness and EMT phenotypes in gemcitabine‐resistant pancreatic cancer cells
Source: J Cell Mol Med. 2017 Feb 28;21(9):2055–67. doi: 10.1111/jcmm.13126 (PMC5571518; doi:10.1111/jcmm.13126)
Supplement: Supplementary file 1 — Table S1 PCR primer sequences. [file JCMM-21-2055-s001.doc]

**Table S1.** PCR primer sequences.

| **Primer** | **Sequence** |
| --- | --- |
| Nanog | | forward: 5’-CCGGTCAAGAAACAGAAGACCAGA-3’ | | --- | |
|  | reverse: 5’-CCATTGCTATTCTTCGGCCAGTTG-3’ |
| Sox2 | | forward: 5’-TCAGGAGTTGTCAAGGCAGAGAAG-3’ | | --- | |
|  | reverse: 5’-GCCGCCG CCGATGATTGTTATTAT-3’ |
| E-cadherin | forward: 5’-AATGCCGCCATCGCTTAC-3’ |
|  | reverse: 5’-CGGAGGATTATCGTTGGTGTC-3’ |
| Vimentin | forward: 5’-CCTGAACCTGAGGGAAACTAATC-3’ |
|  | reverse: 5’-TGCAGAAAGGCACTTGAAAGC-3’ |
| Snail | forward: 5’-GTTTACCTTCCAGCAGCCCTAC-3’ |
|  | reverse: 5’-AGCCTTTCCCACTGTCCTCAT-3’ |
| GLUT1 | forward: 5’-AGAGGTTATGTGCCTGAAGTCG-3’ |
|  | reverse: 5’-GGGTGAAGGAGGAGGATGAG-3’ |
| HKII | forward: 5’-AATTGTCCATGTGCTTCCCTA-3’ |
|  | reverse: 5’-ATCCAGGTTTAATGTCTGTGCTT-3’ |
| LDHA | forward: 5’-ATTTCACTGTCTAGGCTACAACA-3’ |
|  | reverse: 5’-TTAATACCATCCAGCATCAGG-3’ |
| PKM2 | forward: 5’-CCAGCAACGCTTGTAGAACTCA-3’ |
|  | reverse: 5’-GCTGTCACCCTCTTGCCATCT-3’ |
| DCLK1 | forward: 5’-TGATTGAGGAGATGGATGTG-3’ |
|  | reverse: 5’-ACGATGTTCAGGCTATGC-3’ |
| GAPDH | forward: 5’-GACGCTGGGGCTGGCATTG-3’ |
|  | reverse: 5’-GCTGGTGGTCCAGGGGTC-3’ |
