# Supplementary material for: Up‐regulation of glycolysis promotes the stemness and EMT phenotypes in gemcitabine‐resistant pancreatic cancer cells
Source: J Cell Mol Med. 2017 Feb 28;21(9):2055–67. doi: 10.1111/jcmm.13126 (PMC5571518; doi:10.1111/jcmm.13126)
Supplement: Supplementary file 2 — Data S1 Supplementary Materials and Methods. [file JCMM-21-2055-s002.doc]

**Supplementary Materials and Methods**

**Reagents**

Stock solutions of gemcitabine were made every 7 days in 0.9% normal saline and added to the cell culture bottles upon each passaging. 2-Deoxy-D-glucose (2-DG) (Sigma-Aldrich Co., St. Louis, MO, USA) was freshly prepared by dissolving powder into serum-free medium as needed. Hydrogen peroxide (H_2_O_2_) (Amresco, USA) was freshly made by dissolving the original solution in normal saline. N-acetylcysteine (NAC) (Amresco, USA) was also dissolved in normal saline. All chemicals were stored at 4˚C.

**Cell viability assay**

The viability of GR and parental cells was analyzed using a 3-(4, 5-dimethyl-2-thiazolyl)-2, 5-diphenyl-2H-tetrazolium bromide (MTT) assay (Sigma-Aldrich). The half-maximal inhibitory concentration (IC50) was used as a measure of the concentrations needed to reduce the number of cells by half. Cells (6,000/well) were seeded in 96-well plates overnight. The cells were then cultured in the medium (DMEM with 10% fetal bovine serum) containing increasing concentrations of gemcitabine (0.1 μM to 40 μM) combined with or without 2-DG (5 mM), or increasing concentrations of 2-DG (1 mM to 40 mM) for 48 hrs at 37˚C. GR cells were also treated with 2-DG (5 mM) or H_2_O_2_ (200 μM) in the presence or absence of NAC (5 mM) for 24 hrs or 48 hrs. As for the proliferation of the transfected GR cells, cells (2.5 × 10^3^) were seeded and transfected in 96-well plate. The viability was assessed after 2 days and the proliferation was observed for 5 days. Next, 20 μL of MTT (5 mg/mL in phosphate-buffered saline [PBS]) was added and incubated for another 4 hrs. After that, the supernatant of each well was discarded and dimethyl sulfoxide (DMSO) (Sigma-Aldrich) (150 µL) was added. Following agitation for 10 min in the dark on an Eppendorf shaker, absorbance was read at 490 nm in a microplate photometer. Each concentration was tested in 5 replicates. Data were expressed relative to the untreated group which was set as 100% viability.

**Western blot analysis**

Primary antibodies against Nanog (1:1000) (CST, Danvers, MA), Sox2 (1:1000) (CST), E-cadherin (1:500) (Cusa Bio, Wuhan, China), Vimentin (1:1000) (Cusa Bio), Snail (1:1000) (Cusa Bio), GLUT1 (1:1000) (Cusa Bio), HK-II (1:1000) (Cusa Bio), LDHA (1:1000) (Cusa Bio), PKM2 (1:1000) (Cusa Bio), DCLK1 (1:1000) (Proteintech, USA), and GAPDH (1:1000) (CST).

**Flow cytometric analysis**

For determination of pancreatic cancer stem cell markers, cells were washed twice with PBS containing 2% FBS, dissociated into single-cell suspensions, and stained with fluorescein isothiocyanate-conjugated CD24 or phycoerythrin-conjugated CD133 or isotype control IgG antibodies for 30 min at 4˚C. Samples were then washed in PBS containing 2% FBS and analyzed using a flow cytometer. Levels of intracellular reactive oxygen species (ROS) were determined using an ROS assay kit (Beyotime) according to the manufacturer’s instructions. Briefly, cells were collected, washed with PBS, and incubated with dichloro-dihydro-fluorescein diacetate at a final concentration of 10 μM in serum-free medium for 20 min at 37°C. The fluorescence was then measured with a flow cytometer.

**Animal experiment**

**Subcutaneous tumor formation**

GS and GR cells (5 × 10^6^ cells each in 100 μL PBS) were subcutaneously injected in the right flank of BALB/c nude male mice (4-to-5-week-old and 16-20 g in weight of 5 mice/group). In addition, Equal numbers (5 × 10^6^) of GR cells left untreated or treated with 2-DG for 24 hrs were injected into the right flank of nude mice (5 mice/group). The mice were bred in aseptic specific-pathogen-free (SPF) conditions and kept at a constant humidity and temperature (25-28˚C). Tumor growth was monitored twice a week for 3 weeks. Tumors were collected from mice upon autopsy, fixed with 4% paraformaldehyde, embedded in paraffin and cut into 5-μm slices. Immunostaining with an anti-DCLK1 antibody (CST) was performed to detect DCLK1 expression in tumor xenograft specimens.

**Orthotopic transplantation mouse model of pancreatic cancer**

Five-week-old BALB/c male nude mice (20-25 g in weight) were anesthetized with 10% Chloral Hydrate (0.3 mL/100 g) by intra-abdominal injection and a small abdominal incision was made. A number of 5 × 10^6^ cells (GS vs GR cells; GR vs 2-DG-treated GR cells) in 75 μL PBS were injected into the pancreatic head. The injection site was clamped after removal of the syringe. The pancreas was returned to the abdomen and the peritoneum and the skin were closed with continuous suture. All mice were sacrificed 4 weeks after orthotopic implantations. Livers and lungs were inspected for metastases by hematoxylin-and-eosin staining.
